# Complete genome sequence and comparative analysis of two potential probiotics *Bacillus subtilis* isolated from honey and honeybee microbiomes

**DOI:** 10.1186/s43141-020-00050-w

**Published:** 2020-07-22

**Authors:** Abdelhamid A. Hamdy, Mona A. Esawy, Nouran A. Elattal, Magdy A. Amin, Amal E. Ali, Ghada E. A. Awad, Ian Connerton, Nahla M. Mansour

**Affiliations:** 1 Chemistry of Natural and Microbial Products Department, Pharmaceutical Industries Research Div., National Research Centre, 33 El Bohouth St., Dokki, P.O. Box: 12622, Cairo, Egypt; 2 Department of Microbiology and Immunology, Faculty of Pharmacy, Cairo University, Cairo, Egypt; 3 Division of Food Sciences, School of Biosciences, University of Nottingham Loughborough, Sutton Bonington Campus, Leicestershire, LE12 5RD UK; 4 Gut Microbiology & Immunology Group, Chemistry of Natural & Microbial Products Department, Pharmaceutical Industries Research Division, National Research Centre, 33 El Bohouth St., Dokki, 12622, Cairo, Egypt

**Keywords:** Genome sequence, *Bacillus subtilis*, Probiotics, Honey, Honeybee

## Abstract

**Background:**

We have previously isolated *Bacillus subtilis* HMNig-2 and MENO2 strains, from honey and the honeybee gut microbiome respectively, and demonstrated these strains to produce levansucrase with potential probiotics characteristics. Here we report their complete genome sequence and comparative analysis with other and other *B. subtilis* strains.

**Results:**

The complete genome sequences of *Bacillus subtilis* HMNig-2 and MENO2 were de novo assembled from MiSeq paired-end sequence reads and annotated using the RAST tool. Whole-genome alignments were performed to identify functional differences associated with their potential use as probiotics.

**Conclusions:**

The comparative analysis and the availability of the genome sequence of these two strains will provide comprehensive analysis about the diversity of these valuable *Bacillus* strains and the possible impact of the environment on bacterial evolution.

**Significance and impact of study:**

We introduce complete genome sequence of two new *B. subtilis* strains HMNig-2 and MENO2 with probiotic potential and as cell factories for the production of levan and other valuable components for pharmaceutical and industrial applications

## Background

Honey has been used as a preserve and to treat infection for four millennia [1]. Honey is known for its antibacterial activities, which relate to the properties of viscosity, acidity, and the presence of inimical substances such as H_2_O_2_, flavonoids, and phenolic acids [2]. However, it has been documented that natural honey also contains a small number of microorganisms that constitute the indigenous microflora and which have probiotic potential [3] .Thus, we have used honey and the honey bee as sources of levansucrase producing probiotic bacteria. Two candidate probiotic bacteria that produce levansucrase were isolated, HMNig-2 and MENO2, which were identified as *Bacillus subtilis* species [4, 5].

The natural characteristics of honey that include pH, moisture content, antimicrobial components and sugar concentration mark it as a suitable environment for the persistence of spore-forming bacteria. Even though soil is considered the principal habitat for *Bacillus* species, they have been detected in honey [6] and the feces of bee larvae [7, 8].

*Bacillus* strains have been selected for use in probiotic supplements for human and animal consumption [9–12]. As these bacteria form spores, they present advantages over other common probiotic species with the ability to tolerate high temperatures and sustain viability for long periods without the need for cooling or freezing [12, 13]. *Bacillus* species are also widely used in industrial biotechnology as cell factories to produce a multiplicity of metabolites including antibiotics, bioinsecticides, enzymes, and lipopeptides [14–17].

Levansucrase is an extracellular enzyme encoded by the *sacB* gene, which belongs to the glycoside hydrolase (GH) 68 family produced by several Gram-positive and Gram-negative microbial species. In the presence of high sucrose concentrations, *B. subtilis* produces levan by means levansucrase activity. Industrial applications of levan have been proposed for food and pharmaceutical use. In food applications, levan has been used as an emulsifier, formulation aid, stabilizer and thickener, surface-finishing agent, encapsulating agent, and a carrier for flavor and fragrances [18]. In medicine, levan has shown promise as a plasma substitute, drug activity prolongator, and antihyperlipidemic agent [19]. Although a number of microorganisms are capable of producing levan, including *Bacillus polymyxa* [20], *Acetobacter xylinum* [21], *Lactobacillus sanfranciscensis* [22], *Leuconostoc mesenteroides* [23], *Microbacterium laevaniformans* [24], *Zymomonas mobilis* [25], *B. subtilis* [26], *B. amyloliquefaciens* [27], and *Pseudomonas syringae* [28]; relatively few have been demonstrated to produce levan suitable for food use or in yields of necessary to support commercial production. These restrictions have limited the application of levan.

Here we announce the complete genome sequence and the comparative analysis of the levansucrase producing *B. subtilis* strains HMNig-2 and MENO2 which have been isolated from honey and the honey bee microbiomes. The comparative analysis of these two strains and the availability of their genome sequences will provide a basis for their application as probiotics as well as cell factories for the levan production and other valuable components.

## Methods

### Strains and growth conditions

*B. subtilis* strains HNMig-2 and MENO2 are isolated previously in our laboratory from honey samples and honey bee microbiome [4]. *B. subtilis* strains were grown at 37 °C in Luria Bertani (LB) broth or agar plates made by adding 1.5% agar to the LB medium.

### Genomic DNA extraction

The bacterial cultures were subjected to genomic DNA extraction, using AxyPrep bacterial genomic DNA miniprep kit (Axygen Biosciences, Union City, CA, USA) according to the manufacturer’s instructions and the quality checked using a NanoDrop instrument (ThermoFisher, Loughborough, UK) and by 1% agarose gel electrophoresis.

### Genome sequencing

Genome DNA from libraries prepared using the Illumina Nextera^TM^ tagmentation protocol and run on the MiSeq using an Illumina v3 cassette (Illumina, USA). The genome sequences were de novo assembled from approximately 3.2 million 80 to 250-bp paired-end reads each for HMNig-2 and MENO2 using CLC Genomics Workbench v10.01 and v12.03 (Qiagen Bioinformatics, Denmark). DNA sequence contigs were aligned to the rhizosphere sourced *B. subtilis* strains GQJK2 (NCBI accession CP020367) and TLO3 (NCBI accession CP021169) using whole-genome alignment within CLC Genomics Workbench and used in support of genome sequence assembly.

### Genome annotation and comparison

The complete genomes of *B. subtilis* strains HMNig-2 and MENO2 were annotated using the RAST server [29]. The CGView Comparison Tool was used to generate the maps [30] using BLAST comparisons with an Expect value of 0.1. The first and second rings show the locations of protein coding, tRNA, and rRNA genes on the forward and reverse strands, respectively. The black plot depicts GC content with the peaks extending towards the outside of the circle representing GC content above the genome average, whereas those extending towards the center mark segments with GC content lower than the genome average. The innermost plot depicts GC skew, and both base composition plots were generated using a sliding window of 50,000 nt. The complete genome sequences of the two *Bacillus subtilis* strains have been deposited in the GenBank database.

### Phylogenetic and comparative analysis of levansucrase production

The nucleotide and amino acid sequences encoded by the levansucrase operon from the *B. subtilis* isolates were analyzed by using the BLAST server against the Swiss-Prot protein database [http://www.expasy.org/tools/blast/], and the sequences aligned with the CLUSTALW interface in MEGA7.0 (http://www.megasoftware.net/; pairwise alignment gap opening penalty, 10; gap extension penalty, 0.1; multiple alignment gap opening penalty, 10; gap extension penalty, 0.2). A structure-based alignment was performed with MUSCLE, and the resulting alignment was further refined manually.

## Results

### General genome features

The results of the genome sequence of the two strains indicate similarities but they differ in size and coding capacity. Their general genome features are presented in Table 1, which show that the genomes of *B. subtilis* HMNig-2 and MENO2 contain 4,188,942 and 4,084,669 bases predicting 4239 and 4127 CDSs respectively, and with average of GC contents of 43.6 and 43.8% respectively.

**Table 1:** General genomic features of *Bacillus subtilis* strains

Feature	*Bacillus subtilis* HMNig-2	*Bacillus subtilis* MENO2
Genome size (bp)	4,178,124	4,083,694
L50	1	1
GC content %	43.6	43.8
Number of subsystems	476	473
Number of coding sequences	4239	4127
Number of RNAs	129	128

Comparison with database genome sequences of *Bacillus* species revealed that they exhibit 85–95% DNA sequence identity between the CDSs present in these strains and many *B. subtilis* strains. The complete genome sequences for the two strains are available in GenBank and assigned the nucleotide accession numbers CP031784 for HMNig-2 and CP031783 for MENO2.

### Comparative study of the *B. subtilis* HMNig-2 and *B. subtilis* MENO2 genomes

**Fig. 1:**
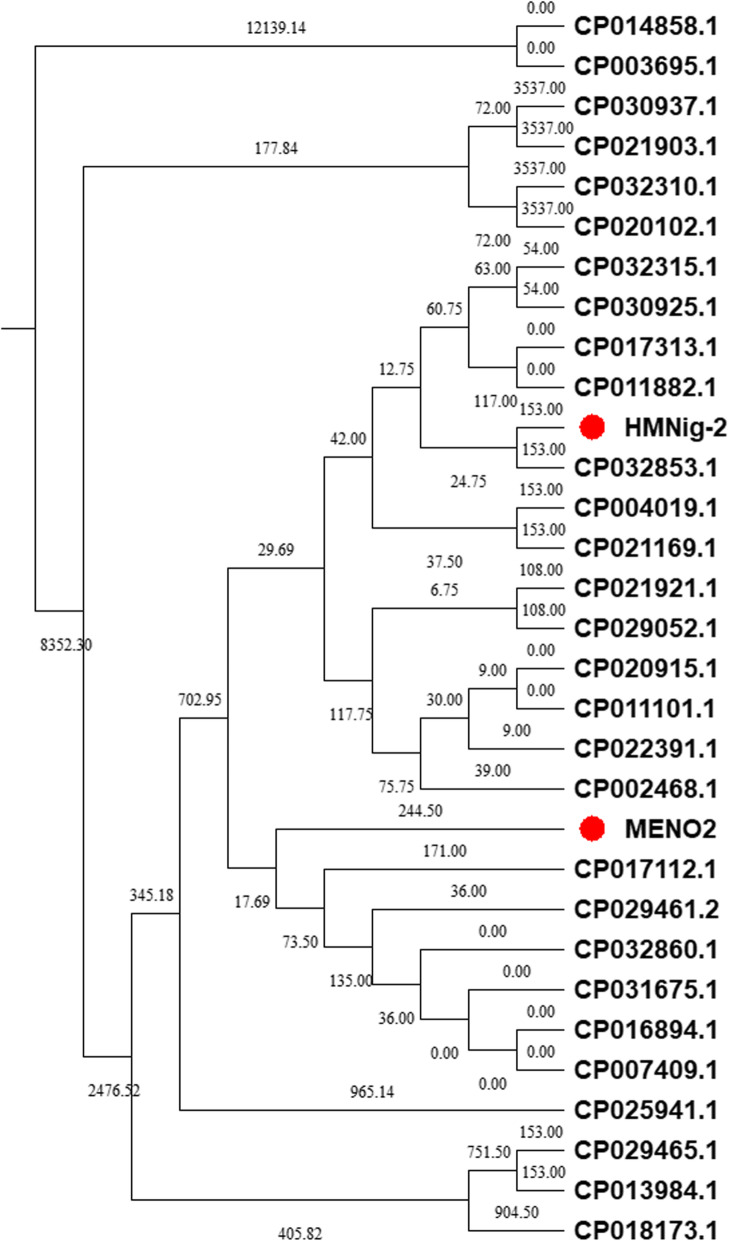
Neighbor-joining phylogenetic tree of the *B. subtilis* HMNig-2 and MENO2 genome, their closest neighbors from other *B. subtilis* strains are shown. Accession numbers are given in parentheses. Phylogenetic analysis was performed using MEGA7

**Fig. 2:**
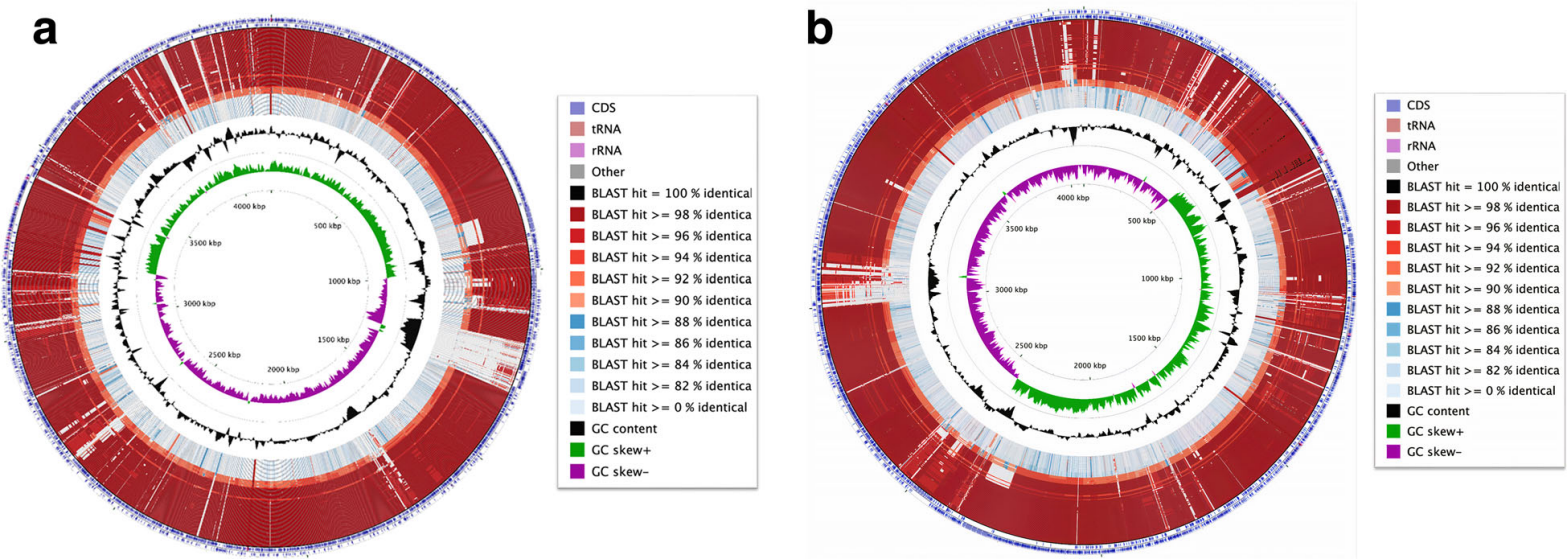
**a** Visualizing features, ORFs, start and stop codons of *B. subtilis* HMNig-2 genome, and comparing the sequence with proteins encoded by other *B. subtilis*. This map was generated by CGview. The first and second [outermost] rings show the locations of protein coding, tRNA, and rRNA genes on the forward and reverse strands, respectively. All subsequent rings, with the exception of the two innermost plots, display regions of similarity detected using BLAST [*E* value = 0.1] between the HMNig-2 genome sequence and the genome sequences of several related sequences [*n* = 100]. Regions of similarity are colored based on the percent identity between the aligned sequence segments. The black plot depicts GC content with the peaks extending towards the outside of the circle representing GC content above the genome average, whereas those extending towards the center mark segments with GC content lower than the genome average. The innermost plot depicts GC skew. Both base composition plots were generated using a sliding window of 50,000 nt. **b** Visualizing features, ORFs, start and stop codons of *B. subtilis* MENO2 genome, and comparing the sequence with proteins encoded by other *B. subtilis*. This map was generated by CGview. The first and second [outermost] rings show the locations of protein coding, tRNA, and rRNA genes on the forward and reverse strands, respectively. All subsequent rings, with the exception of the two innermost plots, display regions of similarity detected using BLAST [*E* value = 0.1] between the MENO2 genome sequence and the genome sequences of several related sequences [*n* = 100]. Regions of similarity are colored based on the percent identity between the aligned sequence segments. The black plot depicts GC content with the peaks extending towards the outside of the circle representing GC content above the genome average, whereas those extending towards the center mark segments with GC content lower than the genome average. The innermost plot depicts GC skew. Both base composition plots were generated using a sliding window of 50,000 nt

The complete genomes of HMNig-2 and MENO2 were analyzed by genome Blast to find the closet neighbors for each strain. From these results, phylogenetic trees were generated which are presented in Fig. 1. The complete genomes of HMNig-2 and MENO2 were compared to 100 of the closest *B. subtilis* strains available to detect regions of similarity among them. These results are shown in Fig. 2a and b.

#### Levansucrase gene

**Fig. 3:**
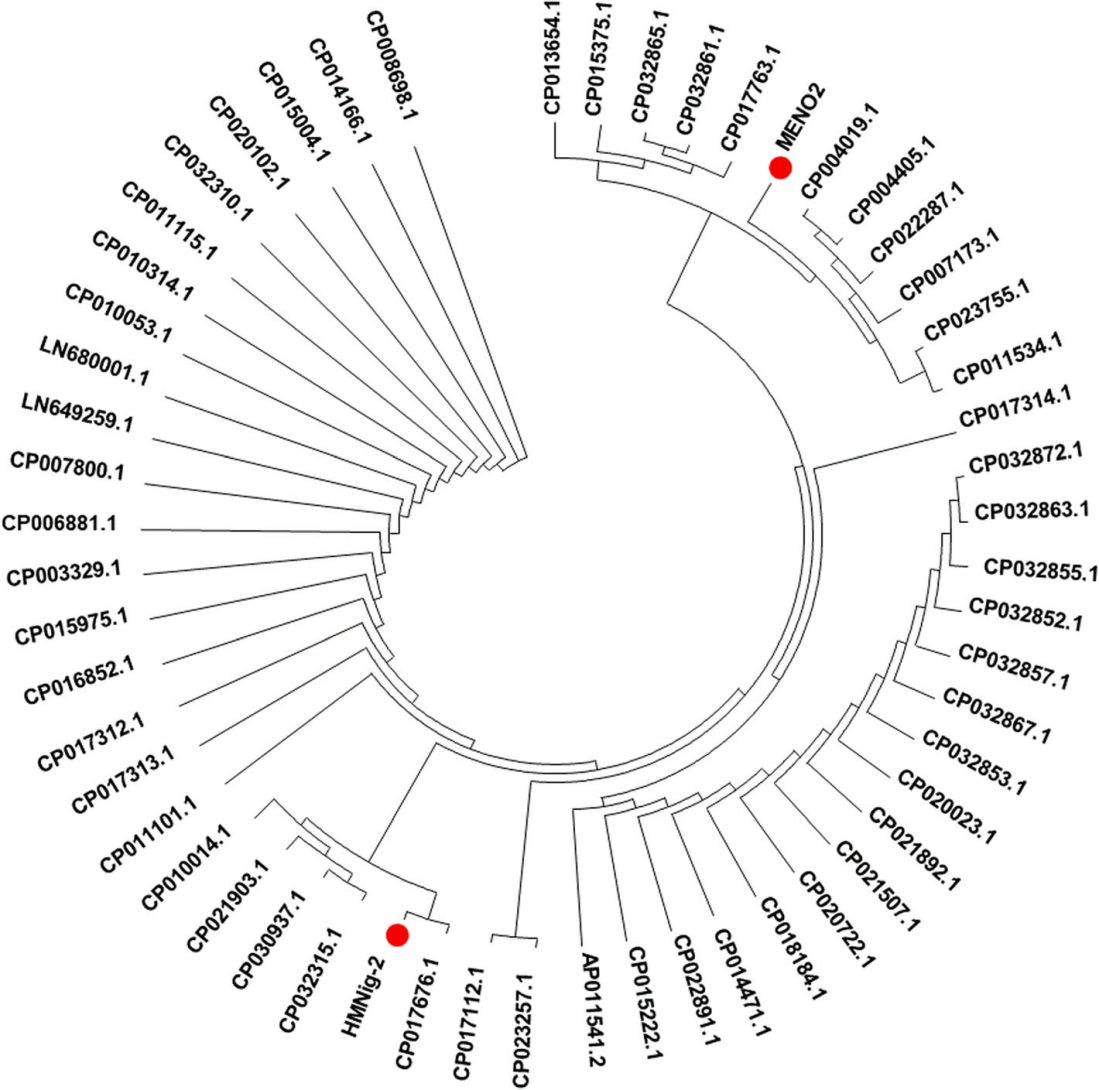
Phylogenetic analysis based on the levansucrase encoded by *sac*B gene, their closest neighbors from other *B. subtilis* strains are shown. Accession numbers are given in parentheses. Phylogenetic analysis was performed using MEGA7

The nucleotide sequences of the levansucrase genes encoded by *sac*B were identified in the genome sequences of HMNig-2 and MENO2 as ORFs of 1419 bp and 1417 bp that code for proteins of 473 and 472 amino acids respectively. The nucleotide sequences of the *sac*B genes were aligned with those of *B. subtilis* isolates from different sources (soil, food, gut, sea, plant) using clustal Omega, and from which a phylogenetic tree was generated (Fig. 3). The phylogenetic tree is formed of five clades, although the sources of isolation do not coincide with the clade distributions, and notably HMNig-2 and MENO2 are not located in the same clade. BLAST searches at NCBI for these sequences at the nucleotide and amino acid sequence level showed high sequence identity with *sac*B from Gram-positive microorganisms such as *B. subtilis* (99%) (strains: ge28, VV2, YP1, TLO3, and BS16045), *B. licheniformis* strain SRCM101441 (98%), *B. amyloliquefaciens* (83%) (strains: LM2303, L-S60, L-H15, LFB112, and Y14), and *Lactobacillus reuteri* (41%) (accession numbers: ABQ01720.1 and AVK93185.1). The sequences presented show reduced identity to *sac*B sequences from Gram-negative microorganisms including *Zymomonas mobilis* strains (26%) (WP_013934123.1, WP_011240294, AAG29870.1, AAV88999.2) and *Erwinia amylovora* (26%). The amino acid sequences of *sac*B gene from *B. subtilis* HMNig-2 and MENO2 showed high similarities (99%) to glycoside hydrolase family 68 protein of *B. subtilis* and multispecies strains (accession numbers: WP_069703641.1, WP_086344351.1, WP_032726907.1, WP_087987552.1, WP_015251171.1, WP_014115253.1, WP_024571701.1). High sequence identity was also noted for the amino acid sequences of *B. subtilis* levansucrase for which crystallographic structures are available (PDB accession numbers: 1OYG_A, 3BYJ_A, 3BYK_A) and *B. subtilis* levansucrase of biotechnological use (accession numbers: KIU05730.1, KFI02859.1, AAN75494.1, CBI68350.1).

The levansucrase sequences of both HMNig-2 and MENO2 exhibit the key features of the *Bacillus* enzymes: a signal peptide MNIKKFAKQATVLTFTTALLAGGATQAFA corresponding to residues 1-29, 3 active sites residues corresponding with D86, D247, and E342. In addition, they have a conserved residue at D339, which is known to bind Ca^2+^ in *B. subtilis* levansucrase. The levansucrase reading frame of HMNig-2 had 473 amino acids in common with other *B. subtilis* levansucrase sequences but contained two amino acid substitutions at S70P and D203N. The MENO2 sequence featured a single substitution at K174H and was missing the final lysine residue common in *B. subtilis* levansucrase proteins.

### Comparative analysis between *B. subtilis* HMNig-2 and *B. subtilis* MENO2

#### Cell wall and capsule

Wall teichoic acids are anionic, phosphate-rich polymers related to the peptidoglycan of Gram-positive microorganisms. In the *B. subtilis* type strain 168, the predominant wall teichoic acid type is poly(glycerol phosphate) while it is poly(ribitol phosphate) in strain W23, where they are synthesized by the *tag* and *tar* gene products, respectively. Cell wall teichoic acids are essential in *B. subtilis* but could be replaceable under phosphate-limiting conditions. The putative poly ribitol phosphotransferase and ribitol-5-phosphate cytidylyl transferase genes are present in *B. subtilis* HMNig-2 but absent in *B. subtilis* MENO2, while three further genes encoding components of the same subsystem, CDP-glycerol:poly (glycerophosphate) glycerol phosphotransferase (EC 2.7.8.12), minor teichoic acid biosynthesis protein *Gga*A, and minor teichoic acid biosynthesis protein *Gga*B, are present in MENO2 but absent in HMNig-2.

#### DNA metabolism

Within this category and under the subsystem DNA repair genes, bipolar DNA helicase HerA, hypothetical protein, ATP-dependent DNA ligase, DNA-cytosine methyltransferase, very-short-patch mismatch repair endonuclease (G-T specific), and protease III precursor are present in the HMNig-2 strain but are absent in MENO2. While MENO2 strain has a prophage-associated gene responsible for recombinational DNA repair in RecT, which is absent from the HMNig-2 strain, recombinational DNA repair is closely tied to cellular replication systems, which functions to repair impairments at replication forks.

#### Cofactors, vitamins, prosthetic groups, and pigments

The gene encoding butyryl-CoA dehydrogenase is present within HMNig-2 strain but absent from MENO2. Butyryl-CoA dehydrogenase in *B. subtilis* is placed within the folate and pterin subcategory.

#### Membrane transport

Two genes encoding CopC and CopD of the copper transport system [31] are present in strain MENO2 but are absent in HMNig-2 strain. This could be an indication of a requirement of the MENO2 strain to adapt to changes in copper availability, either when limited or to avoid toxicity.

#### Nitrogen metabolism

Within the subsystem for ammonia assimilation the gene encoding glutamine amidotransferase, class-II is present in MENO2 but missing from the honey HMNig-2 strain. Glutamine phosphoribosylpyrophosphate (PRPP) amidotransferase is the key regulatory enzyme of de novo purine nucleotide synthesis. Phosphoribosylamine is transformed in a series of steps to the purine nucleotide IMP, which is converted to the major end products of the pathway AMP and GMP. This enzyme is subject to tight metabolic regulation in *B. subtilis*.

#### Phages, prophages, transposable elements, and plasmids

Phage-associated metabolic functions encoding thioredoxin and DNA helicase were observed in the genome of HMNig-2 but absent from strain MENO2, whereas the genes encoding the structural phage neck proteins are present from MENO2 and absent from HMNig-2 genome.

#### Protein metabolism

The large subunit ribosomal protein L14p (L23e) was absent from MENO2. The gene for the tRNA proofreading protein STM4549 was absent from HMNig-2. The protein processing and modification subsystem include N-linked glycosylation in bacteria, and specifically, the gene encoding a putative 4-keto-6-deoxy-*N*-acetyl-d-hexosaminyl-(lipid carrier) aminotransferase was absent from HMNig-2

#### Virulence, disease, and defense

The genes encoding for virulence factors such as hemolysin BL, non-hemolytic enterotoxin NHE, enterotoxin T, cytotoxin T, and cereulide were not present in either of the two strains. Furthermore, their genomes do not contain plasmids or the antibiotic-resistant genes against fluoroquinolone, fosfomycin, streptogramin B, nitroimidazole, phenicol, rifampicin, sulphonamide, and tetracycline.

## Discussion

*Bacillus* spp. have been comprehensively studied due to their tough stress resistance and exceptional environmental flexibility. *B. subtilis* is a model Gram-positive bacterium that has been widely studied for research and biological purposes. It is widespread in nature and safe for humans and the environment; in addition, it has a variety of antimicrobial components and beneficial enzymes for biocontrol and pharmaceutical applications. Moreover, several strains of *B. subtilis* have been identified as probiotic candidates, and it is well known that the probiotic action is strain-specific. The *B. subtilis* HMNig-2 and MENO2 strains were previously isolated from honey and the honeybees gut microbiomes [4]. Our previous results showed their ability for high levansucrase production and levan yield. They also exhibited probiotic characteristics in vitro by expressing tolerance to gastrointestinal conditions. Furthermore, the two strains and their levans showed effects on the immune system and protection from the *Salmonella typhimurium* infection when administrated to mouse groups, suggesting them as promising probiotic candidates [5].

Here in this work, we introduce the complete genome sequences of these valuable strains as probiotic and/or cell factory. The availability of the complete genome sequence of HMNig-2 and MENO2 not only will promote upgrade the genome database, but will also provide the opportunity to explore more genes related to different industries.

Whole-genome sequencing of various *Bacillus* strains has revealed adaptive functions in their gene contents to different environments [32–34]. In this paper, we have identified differences between our two strains HMNig-2 and MENO2, suggesting differential adaptation to the prevailing environmental conditions.

In recent years, many studies have focused on microbial sources of levansucrase (SacB), and in particular the enzyme from *B. subtilis* to reveal an important issue concerning the production of high molecular weight levan that is preferred for multiple applications [35]. It is reported that the molecular weight of the levan produced is affected by the concentration of the enzyme used in the reaction [36, 37]. The authors demonstrate that using low enzyme concentrations produces high molecular weight of levan (HMW) with an average molecular weight of 2300 kDa, whereas higher enzyme concentrations produced a low-molecular weight (LMW) levan of 7.2 kDa. *Bacillus* levansucrase supports two mechanisms dependent on the enzyme concentration, a processive reaction at low enzyme concentrations leading to HMW levan and a non-processive reaction at high enzyme concentrations that produces LMW levan [36]. Protein-product interactions are thought to be responsible for the ensuing mechanism [36], and variation in the amino acid residues responsible for these interactions will influence levan production. Therefore, strain-dependent variation in the *sacB* gene has the potential to modulate levan production.

In the other hand, with respect to their use as probiotics, they approved their safety by the absence of any virulence and antibiotic-resistant genes containing plasmid. Also, the genes encoding for virulence factors such as hemolysin BL, non-hemolytic enterotoxin NHE, enterotoxin T, cytotoxin T, and cereulide were not present in either of the two strains. Recently, the whole-genome sequencing has been used for the safety assessment of potential probiotic strains and prediction of the presence or absence of genes associated with virulence and adverse metabolite [38–40]. Therefore, in future, the complete genome sequencing would be a crucial and accurate approach for evaluating the safety and control the quality of probiotic strains.

## Conclusions

In conclusion, the complete genome sequences of the prospective probiotic *B. subtilis* strains HMNig-2 and MENO2 isolated from honey and honeybee gut microbiome are reported. The strains are suitable for use as probiotics or cell factories as they do not contain any virulence or antibiotic resistance genes.

## Data Availability

All data generated or analyzed during this study are included in this published article.

## Abbreviations

*B. subtilis*: *Bacillus subtilis*
SacB: Levansucrase
CDS: Coding sequence
ORF: Open reading frame
kDa: Kilodaltons
